# Supplementary material for: Double Valorization for a Discard—α-Chitin and Calcium Lactate Production from the Crab Polybius henslowii Using a Deep Eutectic Solvent Approach
Source: Mar Drugs. 2022 Nov 16;20(11):717. doi: 10.3390/md20110717 (PMC9695577; doi:10.3390/md20110717)
Supplement: Supplementary file 1 [file marinedrugs-20-00717-s001.zip › marinedrugs-2013088-supplementary.pdf]

Supplementary Materials:

Double Valorization for a Discard—α-chitin and Calcium Lactate Production from the Crab *Polybius henslowii* Using a Deep Eutectic Solvent Approach

Colin McReynolds [1,2,†], Amandine Adrien [1,2,†], Arnaud Petitpas [1,2], Laurent Rubatat [1] and Susana C. M. Fernandes [1,2,*]

[1] Universite de Pau et des Pays de l'Adour, E2S UPPA, IPREM, CNRS, 64 600 Anglet, France; c.mc-reynolds@univ-pau.fr (C.M.); amandine.adrien@univ-pau.fr (A.A.); arnaud.petitpas@univ-pau.fr (A.P.); laurent.rubatat@univ-pau.fr (L.R.)
[2] MANTA—Marine Materials Research Group, Universite de Pau et des Pays de l'Adour, E2S UPPA,
64 600 Anglet, France
* Correspondence: susana.fernandes@univ-pau.fr
† These authors contributed equally to this work.

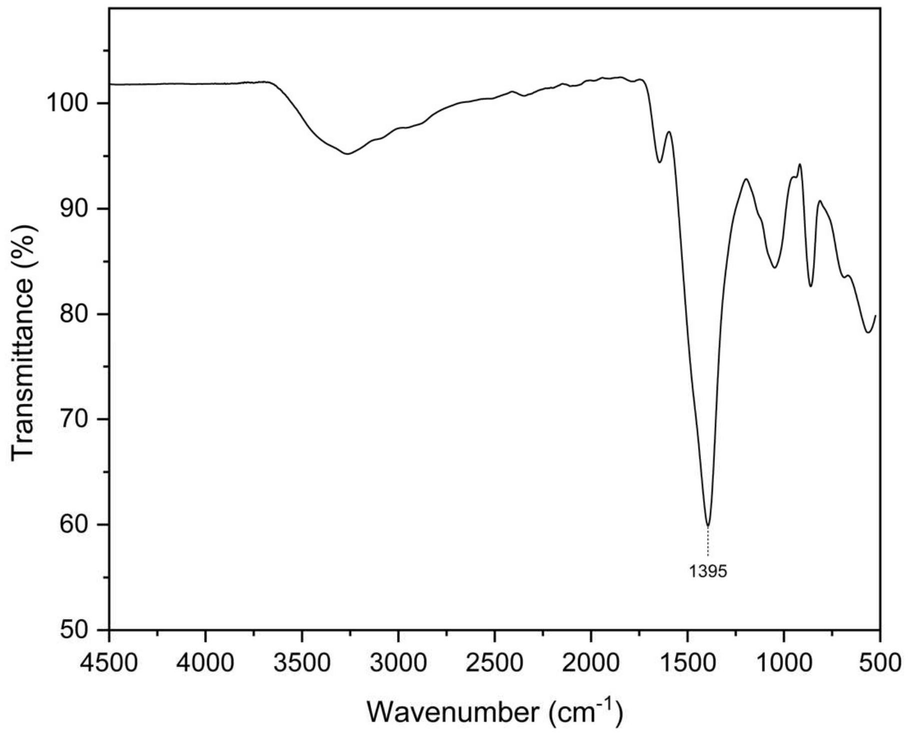

Figure S1. ATR-FTIR spectrum of raw *P. henslowii* carapace.

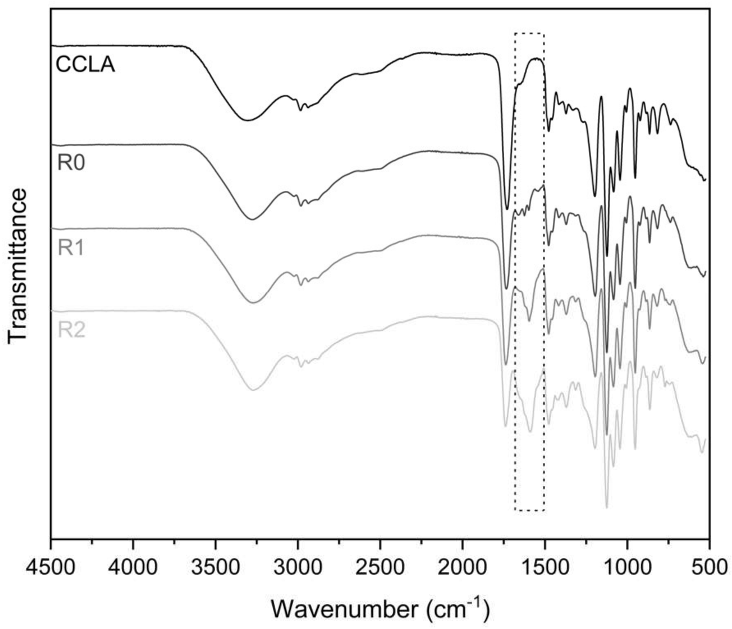

Figure S2. ATR-FTIR spectra of DES post-extraction and ethanol washing. The doted rectangle is included to emphasize where major changes occurred.
